# DNA N6-Methyladenine (6mA) Modification Regulates Drug Resistance in Triple Negative Breast Cancer

**DOI:** 10.3389/fonc.2020.616098

**Published:** 2021-02-03

**Authors:** Xianneng Sheng, Jinqiu Wang, Yu Guo, Jiabo Zhang, Jin Luo

**Affiliations:** Department of Breast and Thyroid Surgery, Ningbo First Hospital, Ningbo, China

**Keywords:** triple negative breast cancer (TNBC), 6mA, MDA-MB-231, Olaparib resistance, LINP1

## Abstract

Triple negative breast cancer (TNBC) is a subtype of breast cancer with strong aggressiveness and poor clinical treatment effect, accounting for about 10–20% of breast cancer cases. N(6)-methyldeoxyadenosine (6mA) is the most conservative DNA modification in prokaryotes and eukaryotes. It is widely found in bacteria and has such functions as DNA mismatch repair, chromosome separation and virulence regulation. We determined that 6mA was modified in TNBC cell line MDA-MB-231 and the TNBC tissue. Meanwhile, compared with normal tissues, the expression level of 6mA and its methylase N6AMT1 was significantly decreased in TNBC tissue. MDA-MB-231cells were cultured with 8μM Olaparib for 2 months to construct drug-resistant cell line 231-RO. It was found that the level of 6mA also increased significantly, and the expression of N6AMT1 or ALKBH1 could effectively influence the drug resistance. Subsequently, we found that LINP1 was highly expressed in 231-RO, which was involved in DNA repair, and the expression of LINP1 could be positively regulated by 6mA modification. LINP1 expression level is directly related to TNBC drug resistance. The above results indicate that 6mA may be a new biological marker of TNBC. Meanwhile, 6mA modification may be involved in the regulation of Olaparib resistance.

## Introduction

Breast cancer, worldwide, is one of the most common types of cancer in women, and it also leads to the highest number of deaths in women aged 20–59 (1). TNBC accounted for 10.0~20.0% of all the pathological types of breast cancer, with a poor prognosis compared with other types of cancer and special biological behavior and clinicopathological characteristics (2, 3). Immunohistochemistry of TNBC showed that estrogen receptor (ER), progesterone receptor (PR) and proto-oncogene HER-2 were negative (2–5). Chemotherapy is the main way to treat TNBC, however, resistance to cytotoxic drugs often leads to treatment failure and death. Therefore, the understanding of the mechanism of drug resistance and effective new treatment strategies become urgent clinical needs.

Poly ADP-Ribose polymerase (PARP) is a DNA repair enzyme. It plays an important role in DNA damage repair and apoptosis (6). To date, several inhibitors of PARP (PARPi) have been applied in clinical cancer treatment with promising results. Olaparib is a highly effective PARPi approved by FDA for clinical application (7), which can treat a variety of cancers including breast cancer (8–10), especially for cancers containing BRCA1/2 mutation with better therapeutic effect (11, 12). However, the emergence of drug resistance also makes the application of these drugs in a difficult situation.

The 6mA modification on DNA is widely present in the genomes of prokaryotes and regulates the functions of bacterial DNA replication, repair, transcription, and bacterial resistance (13). Recent studies have found that 6mA also exists in eukaryotes and plays a regulatory role in DNA transcription and other functions (14–16). Data showed that 6mA was involved in the occurrence and development of tumors and had an impact on tumor progression (16, 17). Based on the above conclusions, we concluded that 6mA May play an important role in TNBC resistance.

In this study, we first collected and detected the modification level of 6mA in TNBC, and compared it with normal tissue to determine the change trend of 6mA. By constructing the Olaparib resistance model, we explored the relationship between 6mA modification and drug resistance. We then preliminarily explained the mechanism of 6mA regulating TNBC resistance generation by regulating corresponding proteins.

## Materials

### Cell Culture

TNBC cell line MDA-MB-231 was purchased from ATCC. MDA-MB-231 was cultured in RPMI1640 medium (Gibco) containing 10% fetal bovine serum (FBS, Gibco). When resistant cell lines were constructed, the screening medium (Basal medium supplemented with 8 μM Olaparib) was changed every 48 h and the culture was continued for 2 months for detection.

### Cell Proliferation Assay

The cells to be tested were seeded in 24-well plates at a density of 25,000 cells per well. After 24 h of culture reached logarithmic growth stage, the culture medium was replaced with experimental medium, and continuously processed for another 48 h. Cell count is measured by manual counting with Cell Counting instrument or by Cell Counting Kit-8 (Beyotime, C0038). Three biological replicates were performed for each set of experiments.

### DNA Extraction and Dot Blot

Tissue samples or cells were homogenized in a lysis buffer with protease K (Sigma-Aldrich) and digested overnight in a 56°C metal bath. On the second day, the samples were treated with RNase A for 12 h, the DNA was purified and dissolved with 10 mM Tris-Hcl (pH 8.0).

After denaturation, purified DNA samples were dripped onto membranes (Amersham Biosciences) according to the experimental design, heated in a hybrid furnace at 80°C for 30 min and sealed with 5% skimmed milk for 1 h, followed by incubation with an 6mA primary antibody (Active Motif, 61755) at 4°C overnight. On the second day, the secondary antibody was incubated at room temperature for 30 min, and then the 6mA level was detected and quantified. Then we soaked the membrane in methylene blue solution and stained the DNA. To compare the total amount of DNA and use it as a positive control.

### Immunofluorescence

We digested the cells with trypsin to make cell suspension. Drops containing a small number of cells were added to the frozen slide at high altitudes to expose the chromosomes. Then chromosomes fixed with 4%PFA and sealed with 5% BSA at room temperature for 1 h. Primary Antibody of 6mA (Active Motif, 61755) was incubated overnight at 4°C, followed by fluorescent secondary antibody (Invitrogen, A32723), with DAPI (Invitrogen, P36931) marking of chromosomes.

### Q-PCR

Total RNA was extracted from tissue samples or cells using TRIzol (Invitrogen), and 500 mg of total RNA was reverse-transcript using a reverse-transcription kit (Vazyme). The SYBR qPCR kit (Vazyme) was then used to detect the expression level of the target gene. 2^−ΔΔCT^ was used to calculate the relative gene expression. The following primers were used:

GAPDH FW TCGGAGTCAACGGATTTGGT
GAPDH RV TGAAGGGGTCATTGATGGCA
LINP1 FW CCCGAAATTCAAGCCACACA
LINP1 RV TCCCCATACCCTCTCCTACC
ALKBH1 FW CCTGGTGCCCAAAAGGTGAT
ALKBH1 RV TGAGTCCATAGGCTTGCCAC
N6AMT1 FW GCAGCAGCTTGTACCCTAGA
N6AMT1 RV GGTAGCAAGCCTTTGACCAAATC

### Western Blot

Proteins were extracted from tissue or cell samples using RIPA (Abcam). The 20 mg protein was separated by SDS-PAGE and then transferred to the nitrocellulose membrane. A buffer containing 5% skimmed milk powder was used to seal at room temperature for 1 h, followed by incubation with primary antibody overnight. On the second day, secondary antibody was incubated and color and quantitative analysis were performed. GAPDH (Abcam, 1:10,000, ab8245), N6AMT1 (Invitrogen, 1:1,000, PA5-42782), ALKBH1 (Abcam, 1:1,000, ab126596), P53 (Abcam, 1:1,000, ab26), γ-H2AX (Abcam, 1:1,000, ab124781).

### γ-H2AX Resolution Assay

The cells were seeded in a six-well plate and irradiated with 5 Gy when the growth rate reached 80%. The irradiated cells were then collected and used for protein extraction. Western detection for γ-H2AX level.

### The Statistics

All quantitative data was counted and analyzed using Graphpad 5. The data were shown as the percentage of controlled mean ± standard deviation, and were evaluated by the two-tailed, double-sample, and equal variance student T test. P value ≤ 0.05 was considered to have a significant difference.

## Results

### 6mA Is the Biological Marker of Triple Negative Breast Cancer

**Figure 1:**
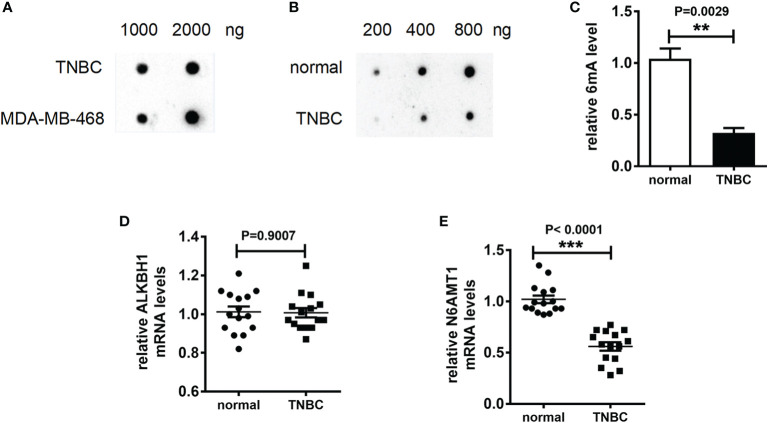
6mA is the biological marker of TNBC. **(A–C)** Dot blot detection of 6mA level; Q-PCR was used to detect mRNA expression levels **(D)** kbh1, **(E)** N6AMt1. **p < 0.01, ***p < 0.001, n = 15.

6mA plays an important role in the life cycle of prokaryotes and is involved in bacterial replication and drug resistance generation. We speculated that 6mA played a specific role in the occurrence of TNBC and had impacted on the regulation of drug resistance. In order to explore the above inference, we collected and extracted DNA from TNBC tissue and TNBC cell line MDA-MB-231. Then we detected the modification level of 6mA in TNBC tissues and TNBC cell lines. The results of Dot blot showed that the 6mA modification on DNA was present in TNBC and TNBC cell line (**Figures 1A**, **S1A**). To confirm this result, we performed immunofluorescence staining on the chromosomes. The result showed that there was a 6mA positive stain on the chromosome (**Figure S1B**). It is worth noting that 6mA levels in TNBC tissues were significantly lower than those in normal tissues (**Figures 1B, C**, **S1C**, p < 0.01). Meanwhile, we found that in the 15 TNBC tissues detected, compared with the control tissues, the expression level of 6mA demethylase ALKBH1 showed no significant trend of change, while that of methylase N6AMT1 showed a significant trend of decrease (**Figures 1D, E**, p < 0.001).

### N6AMT1 Is the Stress Protein From Olaparib That Regulates 6mA Levels

**Figure 2:**
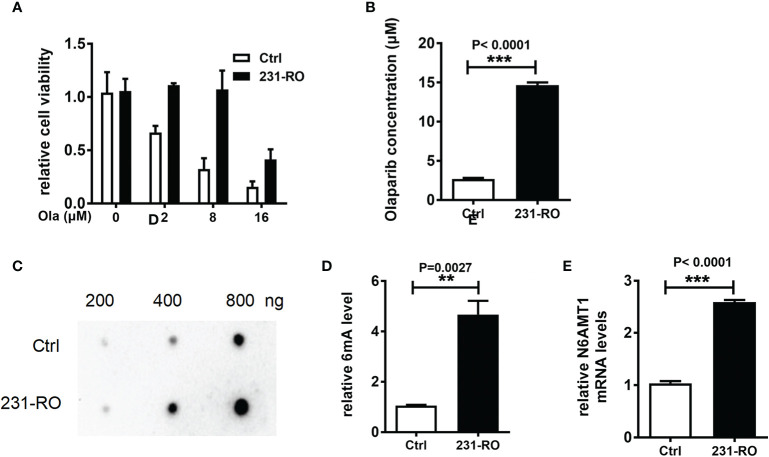
N6AMT1 is the stress protein from Olaparib that regulates 6mA levels. **(A)** Cell survival rate was detected by CCK-8; **(B)** IC50 analysis of drug resistance of Ctrl and 231-RO; **(C, D)** Dot blot detection of 6mA level in each group; **(E)** Q-PCR was used to detect the expression level of N6AMT1. **p < 0.01, ***p < 0.001, n = 3.

In order to explore the relationship between 6mA and TNBC resistance, we constructed the Olaparib resistance cell model. The MDA-MB-231 cell line with Olaparib resistance was cultured for 2 months in a medium containing 8 μM Olaparib, and the MDA-MB-231-RO (231-RO) cell line was obtained. CCK-8 results showed that the survival rate of 231-RO was significantly higher than that of Ctrl (**Figure 2A**, P < 0.001), after 48 h treatment with different Olaparib concentrations. Meanwhile, IC50 results showed that the drug resistance of 231-RO was about six times that of the Ctrl group (**Figure 2B**, P < 0.001).

Then we compared the modifications of the two cell lines, and the results showed that the level of 6mA of 231-RO was higher than that of Ctrl (**Figures 2C, D**, P < 0.01), and methylene blue staining showed no difference in the amount of DNA samples between the two lines (data not shown). Interestingly, the statistics of 6mA modifications in different time periods showed that the trend was first significant rise and then slow decline (**Figure S2A**, n = 3). In line with this, the mRNA level of N6AMT1 also increased significantly (**Figure 2E**, n = 3, P < 0.001), but the expression of ALKBH1 showed no difference (**Figure S2B**, n = 3). In addition to MDA-MB-231 cell lines, we also explored another TNBC cell line (MDA-MB-468), and one ER-positive line (MCF7) as the models for drug resistance experiments. Similar to MDA-MB-231, 6mA of MDA-MB-468 and MCF7 also increased after long-term cultivation with Olaparib, and it was also found that the expression of methylase N6AMT1 also increased. However, both of the above trends were weaker than MDA-MB-231, which may be caused by cell heterogeneity, but their changing trends still showed a high degree of consistency (**Figures S2C, D**).

### 6mA Regulates the Resistance to Olaparib of MDA-MB-231

**Figure 3:**
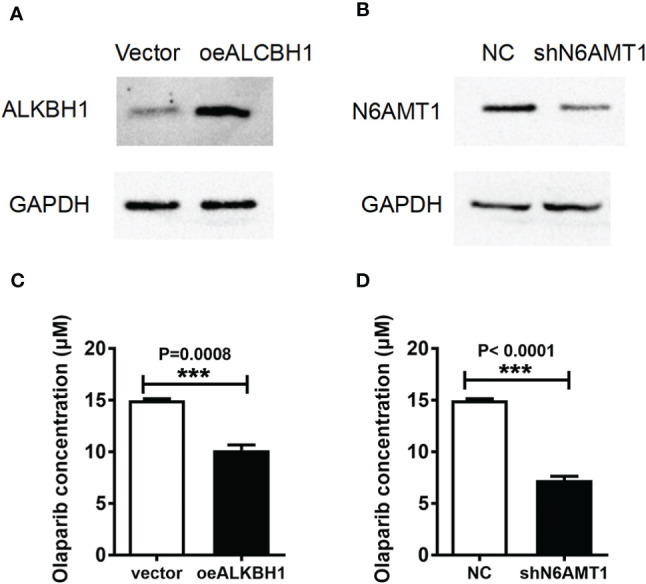
6mA regulates the resistance to Olaparib of MDA-MB-231. The protein expressions of **(A)** ALKBH1 and **(B)** N6AMT1 were detected by Western blot; **(C, D)** IC50 analyzed the changes of drug resistance in each group. ***p < 0.001, n = 3.

In order to determine the effect of 6mA modification on Olaparib drug resistance, we increased ALKBH1 or decreased the expression of N6AMT1 in 231-RO cell lines (**Figures 3A, B**). IC50 results showed that the reverse regulation of both proteins could reduce Olaparib drug resistance (**Figures 3C, D**) and significantly reduce the level of 6mA (**Figure S3A**). Similarly, increasing the expression of N6AMT1 in MDA-MB-231 cell lines can also improve the drug resistance of Olaparib (**Figure S3B**). Since Olaparib is a PARP inhibitor which targets at DNA repair, we speculate that 6mA plays an important role on DNA repair. Then we examined the effects of regulation of 6mA level on DNA damage repair. The expression level of γ-H2AX, a damage marker, after regulating 6mA levels. The results showed that γ-H2AX expression in N6AMT1 overexpressed cells was significantly lower than that in the control group (**Figure S3C**) under the same irradiation condition.

### 6mA Affects Triple Negative Breast Cancer Resistance by Regulating LINP1

**Figure 4:**
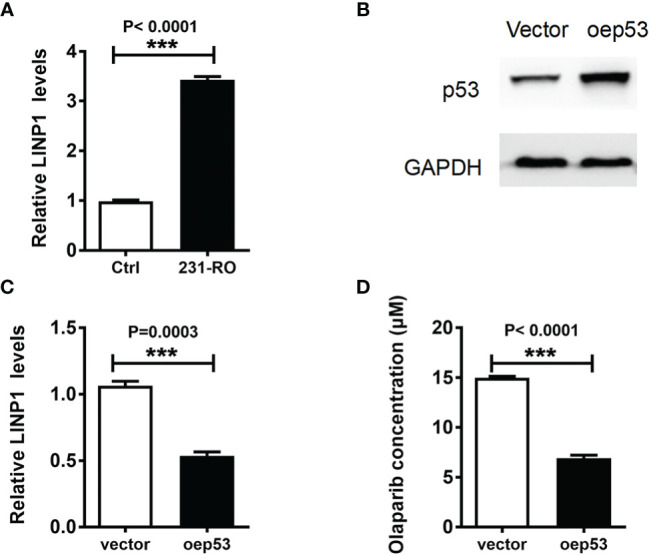
6mA affects TNBC resistance by regulating LINP1. **(A, C)** LINP1 levels was detected by Q-PCR; **(B)** p53 expression level was measured by western blot; **(D)** IC50 analysis the level of Olaparib resistance. ***p < 0.001, n = 3.

Previous studies have shown that 6mA regulates gene expression and shear in eukaryotes (15), especially the transcription of non-coding RNA (18). LINP1 was highly expressed in TNBC (19), and its main function was to enhance DNA damage repair (20). In order to explore the potential mechanism of 6mA affecting the resistance of MDA-MB-231 to Olaparib, we detected the expression level of LINP1 in 231-RO. Compared with MDA-MB-231, the expression level of LINP1 in 231-RO was dramatically increased (**Figure 4A**), while the regulation of 6mA-related proteins positively regulated the expression of LINP1 (**Figures S4A, B**). Subsequently, the expression level of LINP1 was down-regulated by overexpression of p53 protein (20) (**Figures 4B, C**), and changes in 231-RO drug resistance were detected. IC50 results showed that p53 could effectively reduce the resistance of 231-RO to Olaparib (**Figure 4D**).

## Discussion

TNBC has high treatment difficulty and poor prognosis due to its special biological characteristics. However, the emergence of drug resistance also leads to the gradual weakening of the therapeutic effect of TNBC. In the process of exploring the correlation between 6mA and TNBC, we found that the level of 6mA in TNBC samples was lower than that of normal tissues. These results are in contravention of the higher levels of 6mA found by Xie Q et al. in primary Glioblastoma (18), but as the same as Xiao et al. found in primary gastric and liver cancer tissues (17). The opposite trend may be due to tissue heterogeneity, or the different stages of tumor development. Subsequently, our detection results of the expression levels of N6AMT1 and ALKBH1 also showed that 6mA methylase generally had higher expression levels in TNBC tissues. Therefore, the high 6mA should be one of the biological markers of TNBC.

6mA modification is very common in the genomes of prokaryotes, and it is involved in various life activities of bacteria (13). In eukaryotes, 6mA also has a variety of biological functions (21, 22). The TNBC cell lines cultured with 8 μM Olaparib and the cell lines with strong Olaparib resistance were selected. And then, we found that 6mA had a high level in the cell line. We observed that 6mA showed a trend of fluctuation during the screening process. These results indicate that 6mA has a high stress on Olaparib, and the stress also regulates the life characteristics of cells. Q-PCR results suggested that the trend of 6mA was more controlled by N6AMT1, indicating that N6AMT1 might be the stress protein of Olaparib and thus affect the modification level of 6mA. In the subsequent regulation of N6AMT1 and ALKBH1, the variation trend of 6mA modification level consistent with drug resistance was observed. In other words, increasing N6AMT1 or decreasing ALKBH1, respectively, can lead to increased cell resistance to Olaparib, and *vice versa*. This result indicates that changing the 6mA level by regulating different proteins can have expected effects on drug resistance, that is, 6mA can positively regulate the resistance of MDA-MB-231 to Olaparib. Further, it can be inferred that since each protein has multiple functions, the effect of N6AMT1 and ALKBH1 on drug resistance is through the function of regulating 6mA level, rather than other potential protein functions.

LINP1 is an LncRNA highly expressed in TNBC (19), and its main function is to promote DNA repair (20). During the regulation of 6mA, we observed that it had a positive regulatory effect on LINP1 expression level. This phenomenon seems to be contrary to the phenomenon of high expression in TNBC, but in fact it can be inferred that LINP1 is co-regulated by multiple mechanisms. Subsequently, in the regulation of p53 protein, we also found that reducing LINP1 level could restore the sensitivity of 231-RO to Olaparib. This data suggests that LINP1 is indeed a potential mechanism for 6mA to regulate TNBC resistance. It was also shown that LINP1 was regulated by both P53 and 6mA.

In conclusion, we found that 6mA is one of the biomarkers of TNBC. It is also preliminarily proved that N6AMT1 is Olaparib stress protein and can regulate the modification of 6mA. Subsequently, 6mA further regulates the sensitivity of TNBC to Olaparib by regulating the expression level of LINP1. However, there are still many unanswered questions, such as the mechanism by which 6mA regulates the expression of LINP1, and the mechanism by which multiple DNA repair mechanisms work together to make cells develop drug resistance, which still needs further study.

## Data Availability Statement

The original contributions presented in the study are included in the article/**Supplementary Material**; further inquiries can be directed to the corresponding author.

## Ethics Statement

The studies involving human participants were reviewed and approved by the Ningbo First Hospital Ethics Committee (approval no.2019-R046). The patients/participants provided their written informed consent to participate in this study.

## Author Contributions

XS, JW, and YG contributed to the concept design, planning of the study, revision, and final approval of the present article. XS, JZ, and JL are responsible for doing the experiments, writing, analysis, interpretation, revision, and final approval of the present article. All authors contributed to the article and approved the submitted version.

## Funding

This study was funded by the clinical pharmacy project of the Association of Integrated Chinese and Western Medicine of Zhejiang (grant no. 2019LY009).

## Conflict of Interest

The authors declare that the research was conducted in the absence of any commercial or financial relationships that could be construed as a potential conflict of interest.
