# Supplementary material for: DNA N6-Methyladenine (6mA) Modification Regulates Drug Resistance in Triple Negative Breast Cancer
Source: Front Oncol. 2021 Feb 3;10:616098. doi: 10.3389/fonc.2020.616098 (PMC7887291; doi:10.3389/fonc.2020.616098)
Supplement: Supplementary file 1 [file DataSheet_1.pdf]

DNA N6-methyladenine (6mA) modification regulates drug resistance in triple negative breast cancer

Xianneng Sheng[1], Jinqiu Wang[1], Yu Guo[1]*, Jiabo Zhang[1], Jin Luo[1]

1Department of Thyroid and Breast Surgery, Ningbo First Hospital, Ningbo, China.

*email: guoyu308@163.com

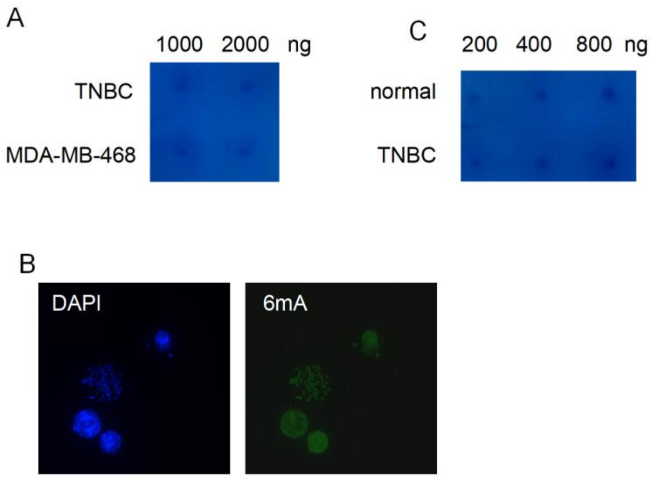

Figure S1 **6mA is the biological marker of TNBC.** A, C. Methylene blue staining labeled DNA in Dot Blot experiment; B. Immunofluorescence labeling 6mA on chromosome, DAPI in blue and 6mA in green.

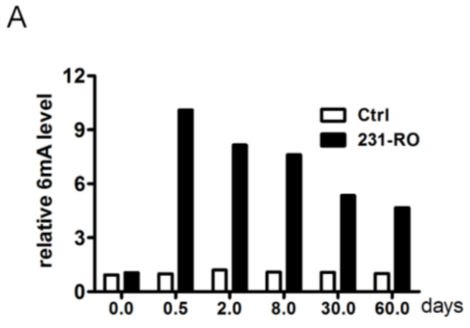

Figure S2 **N6AMT1 is the stress protein from Olaparid that regulates 6mA levels.** A. Dot blot detection of 6mA level in each group; B. Q-PCR was used to detect the expression level of ALKBH1. ***$p < 0.001$, n=3.

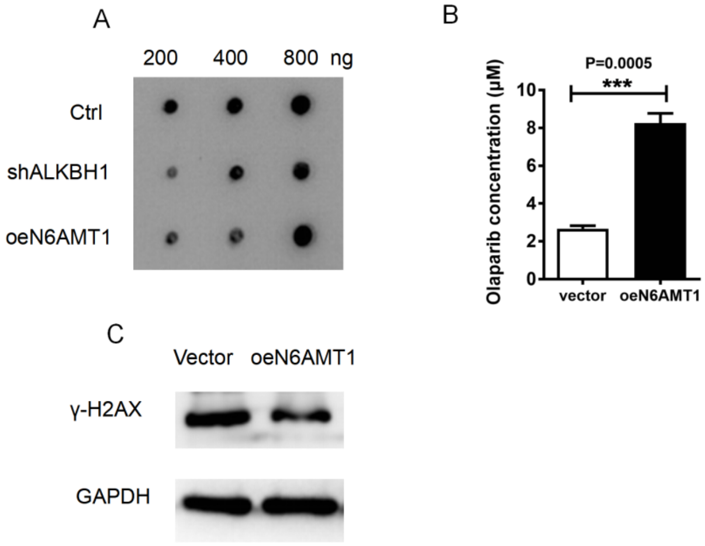

Figure S3 **6mA regulates the resistance to Olaparid of MDA-MB-231.** A. Dot blot detection of 6mA level in each group; B. IC50 analyzed the changes of drug resistance; C. western blot detection for γ-H2AX level. ***p < 0.001, n=3.

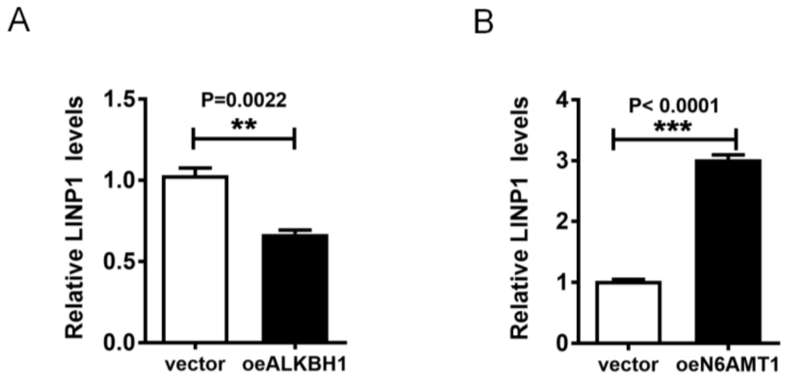

Figure S4 **6mA affects TNBC resistance by regulating LINP1.** A, B. Q-PCR examined the level of LINP1. ***p < 0.001, n=3.
